# New insights into the identity of *Discolaimium dubium* Das, Khan and Loof, 1969 (Dorylaimida) as derived from its morphological and molecular characterization, with the proposal of its transference to *Aporcella* Andrássy, 2002

**DOI:** 10.21307/jofnem-2021-033

**Published:** 2021-03-23

**Authors:** Nasir Vazifeh, Gholamreza Niknam, Habibeh Jabbari, Arezoo Naghavi, Reyes Peña-Santiago

**Affiliations:** 1 Department of Plant Protection, Faculty of Agriculture, University of Tabriz, Tabriz, Iran; 2 Department of Plant Protection, Faculty of Agriculture, University of Maragheh, Maragheh, Iran; 3 Department of Plant Pathology, Faculty of Agriculture, Lorestan University, Khorramabad, Iran; 4 Departamento de Biología Animal, Biología Vegetal y Ecología, Universidad de Jaén, Campus ‘Las Lagunillas’ s/n, Edificio B3, 23071-Jaén, Spain

**Keywords:** *Aporcella*, Iran, LSU-rDNA, Morphology, New combination, Phylogeny, Taxonomy

## Abstract

Three Iranian populations of *Discolaimium dubium* are studied, including their morphological and morphometric characterization, molecular analysis (LSU-rDNA) and the description of the male for the first time. For comparative purposes, this species is distinguished by its 1.10 to 1.40 mm long body, lip region offset by constriction and 8 to 10 µm wide, odontostyle 7.5 to 10.5 µm long with aperture occupying 59 to 76% of total length, neck 300 to 362 µm, pharyngeal expansion 127 to 181 µm long or 44 to 46% of the total neck length, uterus simple and 38 to 53 µm or 1.2 to 1.5 times the body diameter long, *V* = 52 to 58, tail conical (32-38 µm, *c* = 32-43, *c′* = 1.6-2.0) with rounded tip and a hyaline portion occupying 14 to 15% of tail length, spicules 30 to 32 µm long, and two or three widely space ventromedian supplements with hiatus. Both morphological and molecular data support its belonging to the genus *Aporcella*, whose monophyly is confirmed and to which the species is formally transferred as *A. dubia* (Das et al., 1969) comb. n.

In their revision of the genus *Discolaimoides* Heyns, 1963 Das et al. (1969) described *Discolaimium dubium* based on the holotype plus 25 females collected from several European enclaves in Italy, the Netherlands and Switzerland. Later, it was recorded from the Netherlands again (Bongers, 1988), Spain (Liébanas et al., 2003; Peña-Santiago et al., 2005), Hungary (Andrássy, 2009) and India (Sharma, 2011). Thus, it seems to be a component of the nematode fauna inhabiting in northern territories, widely spread out in Europe.

When describing it, Das et al. (1969) characterized *D. dubium* by having, among other features, 1.06 to 1.33 mm long body, lateral chord without conspicuous lateral organs, lip region slightly expanded, odontostyle 8 to 10 µm long with aperture occupying slightly more than one-half of total length, anterior portion of pharynx muscular and enlarging very gradually, absence of *pars refringens vaginae* and conical tail. This was a peculiar combination of traits; therefore, the authors raised a doubt about its identity and named it *dubium*, that is, dubious or doubtful (cf. Andrássy, 2009) and pointed out (p. 486) that ‘… The systematic position of this species is uncertain. Because of general body shape, shape of lips, structure of cuticle and vagina it is included in *Discolaimium*, but it differs from all species of this genus in the thick anterior part of the esophagus, the anterior position of DO and the absence of conspicuous lateral organs. For the moment, however, it cannot be placed elsewhere; the unsclerotized vagina keeps it outside *Eudorylaimus*’. Patil and Khan (1982) were aware of the intricate taxonomy of *D. dubium* and solved the matter with the proposal of the new genus *Neodiscolaimium*, with *N. dubium* as its type and only species. Nevertheless, this action was not followed by other authors (Andrássy, 1990, 2009; Jairajpuri and Ahmad, 1992) who regarded *Neodiscolaimium* as a junior synonym of *Discolaimium* (Thorne, 1939).

Several molecular analyses carried out along the last decade have shown that dorylaimid taxa lacking a conspicuous *pars refringens vaginae*, including discolaims and some aporcelaims, probably share a more recent common ancestor than traditionally assumed (Álvarez-Ortega and Peña-Santiago, 2016; Álvarez-Ortega et al., 2013; Imran et al., 2019; Naghavi et al., 2019).

A general survey conducted during last years to explore the dorylaimid fauna from Iran resulted in the recovering of, among other forms, several populations identified as belonging to *D. dubium*. Their morphological and molecular study should elucidate the evolutionary relationships of this species, and provide new information to understand better the phylogeny of these dorylaimid taxa.

## Materials and methods

### Extraction and processing of nematodes

Several soil samples were collected from depths ranging from 10 to 40 cm, in the active plant root zone of East-Azarbaijan province, northwest Iran, during the period 2016 to 2017. Nematodes were extracted from soil samples using Whitehead and Heming (1965) method, transferred to anhydrous glycerin according to De Grisse (1969), and mounted on glass slides for handling.

### Light microscopy

Measurements were made using a drawing tube attached to an Olympus BX-41 light microscope. The digital images were prepared using a DP50 digital camera attached to the same microscope with differential interference contrast (DIC). Morphometrics included Demanian indices and the usual measurements and ratios. Line illustrations were prepared using CorelDRAW^®^ software version 12. Photographs were edited using Adobe^®^ Photoshop^®^ CS software.

### DNA extraction, PCR, and sequencing

For the molecular phylogenetic studies, DNA samples were extracted from one or two females selected from each population, studied individually on temporary slides, placed on a clean slide containing a drop of distilled water or worm lysis buffer (WLB), and crushed by a sterilized scalpel. Then, the suspension was transferred to an Eppendorf tube containing 25.65 μl ddH2O, 2.85 μl 10 × PCR buffer and 1.5 μl proteinase K (600 μg/ml) (Promega, Benelux, the Netherlands). The tubes were incubated at −80°C (1 hr), 65°C (1 hr) and 95°C (15 min). Each sample was regarded as an independent DNA sample and stored at −20°C until used as polymerase chain reaction (PCR) template. Primers for 28S rDNA D2-D3 amplification/sequencing were forward primer D2A (5´-ACAAGTACCGTGAGGGAAAGTTG-3´) and reverse primer D3B (5´-TCGGAAGGAACCAGCTACTA-3´) (Nunn, 1992). The 25 μl PCR reaction mixture containing 10 μl ddH2O, 12.5 μl PCR master mix (Ampliqon, Denmark), 0.75 μl of each forward and reverse primers and 1 μl of DNA template. PCR was carried out using a BIO RAD thermocycler machine in accordance with Archidona-Yuste et al. (2016). The thermal cycling program for amplification were as follows: denaturation at 94°C for 2 min, 35 cycles of denaturation at 94°C for 30 sec, annealing of primers at 55°C for 45 sec and extension at 72°C for 3 min followed by a final elongation step at 72°C for 10 min. The PCR products were purified and sent for sequencing to Bioneer Company, South Korea. The recently obtained sequences were submitted into the GenBank database under the accession numbers MT079121, MT079122 and MT079123S as indicated on the phylogenetic tree (Fig. 4).

### Phylogenetic analyses

The Basic Local Alignment Search Tool (BLAST) homology search program was used to compare the newly generated sequences with other available sequences in GenBank database. The recently generated sequences were aligned with the other segments of 28S rDNA gene sequences retrieved from the database using MEGA6 software (Tamura et al., 2013). *Paravulvus hartingii* (de Man, 1880; Heyns, 1968) (AY593062) was chosen as outgroup. Bayesian analysis (BI) was performed with MrBayes 3.1.2 (Ronquist and Huelsenbeck, 2003). The best-fit model of nucleotide substitution used for the phylogenetic analysis was selected using MrModeltest 2.3 (Nylander, 2004) with Akaike-supported model in conjunction with PAUP* v4.0b10 (Swofford, 2003). The BI analysis under GTR + I + G model was initiated with general time-reversible model with invariable sites and a gamma-shaped distribution for the 28S rDNA gene was done. After discarding burn-in samples and evaluating convergence, the remaining samples were retained for further analyses. Posterior probabilities (PP) are given on appropriate clades. The tree was visualized and saved using the program Fig tree 1.4.3 v.

## Results

### Systematics

**Figure 1:**
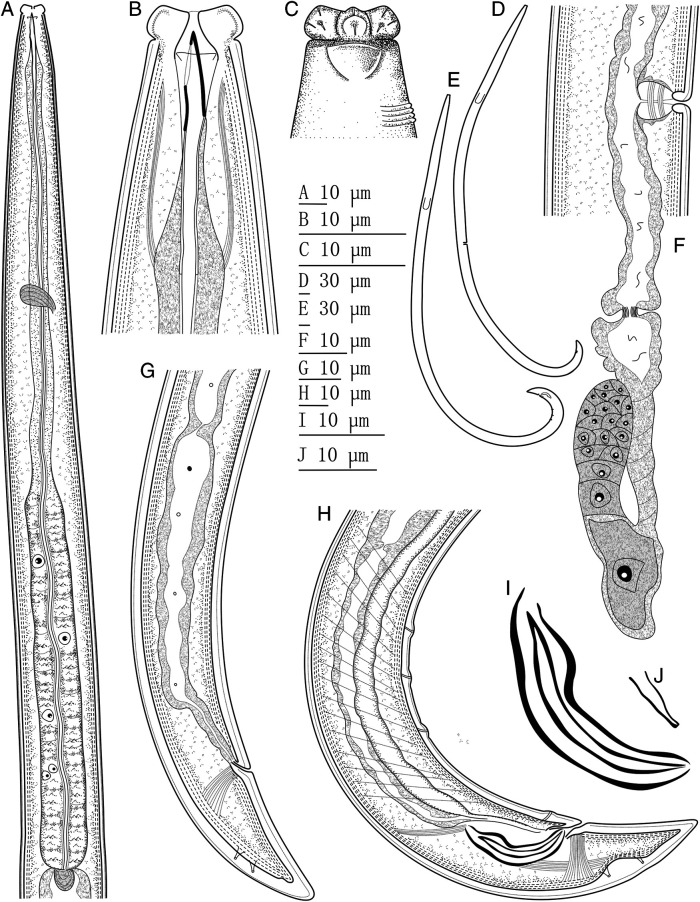
*Discolaimium dubium* Das et al., 1969 from Iran. A: Neck region; B: Anterior region in lateral median view; C: Lip region in lateral surface view; D: Female entire; E: Male entire; F: Female, posterior genital branch; G: Female, posterior body region; H: Male, posterior body region; I: Spicule; J: Lateral guiding piece.

**Figure 2:**
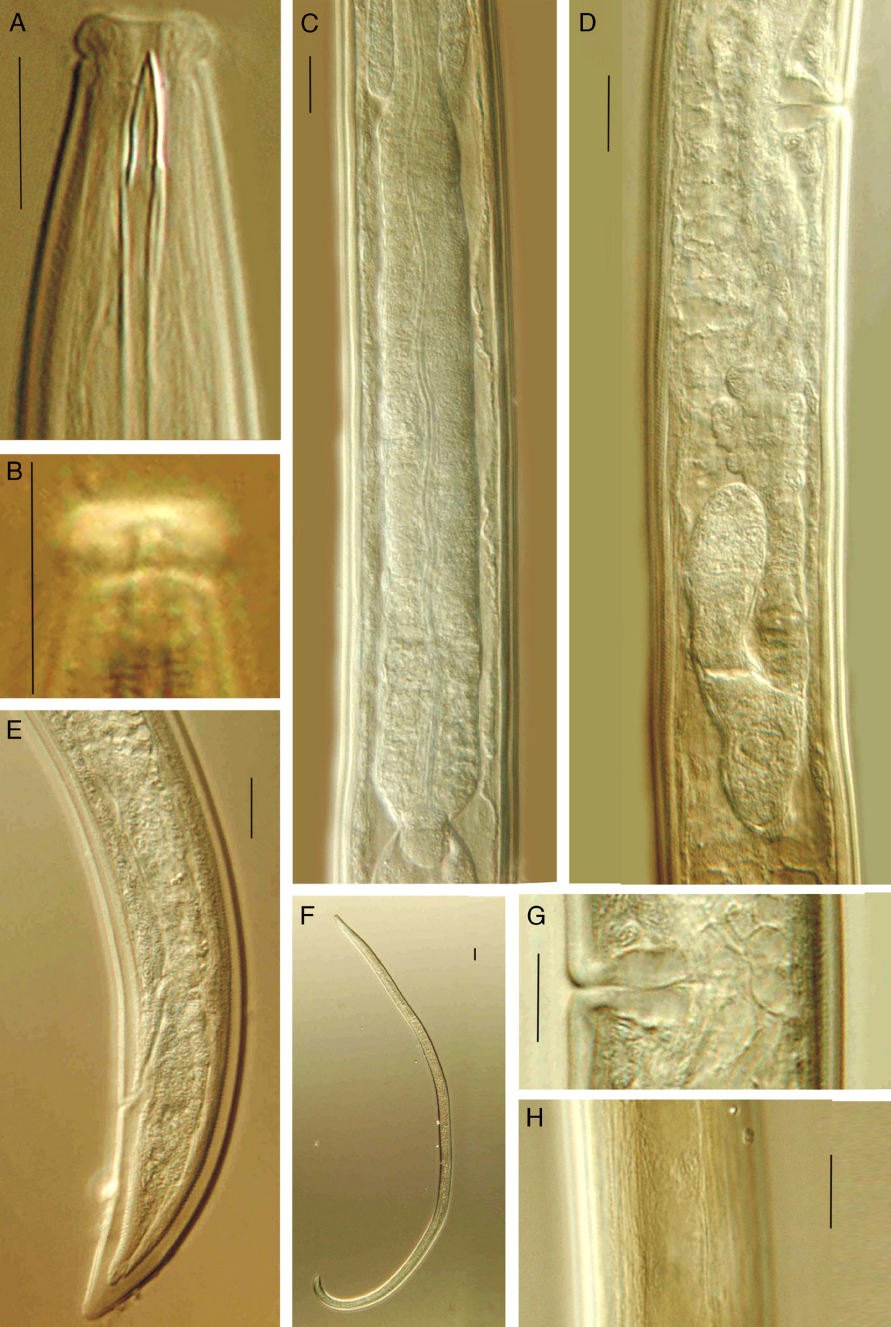
*Discolaimium dubium* Das et al., 1969 from Iran (female, LM). A: Anterior region in lateral median view; B: Lip region in lateral surface view; C: Pharyngeal expansion and pharyngo-intestinal junction; D: Posterior genital branch; E: Posterior body region; F: Entire; G: Vagina; H: Lateral chord. (Scale bars: A-E, G and H = 10 μm; F = 30 μm).

**Figure 3:**
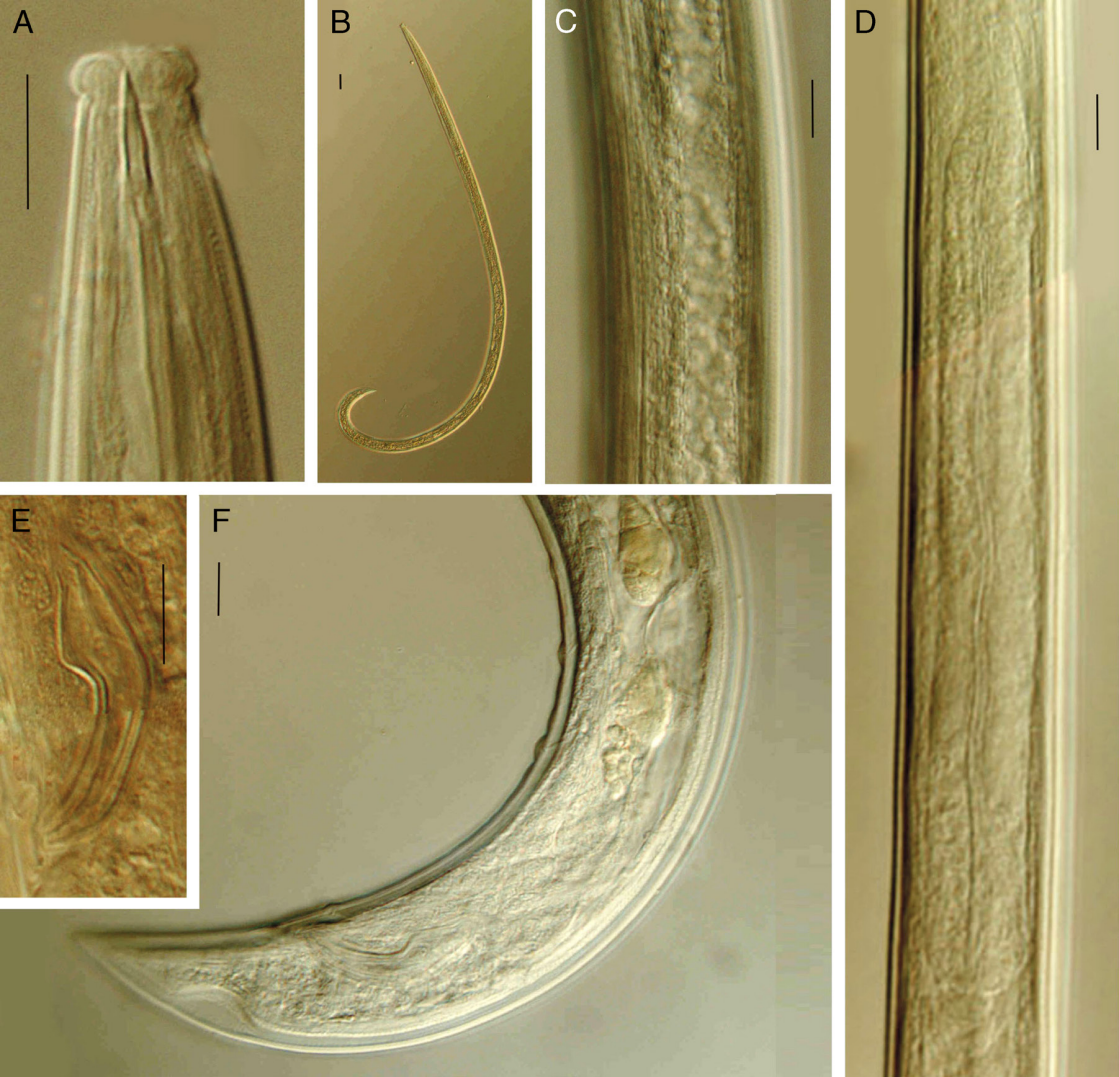
*Discolaimium dubium* Das et al., 1969 from Iran (male, LM). A: Anterior region in lateral median view; B: Entire; C: Lateral chord; D: Pharyngeal expansion and pharyngo-intestinal junction; E: Spicule; F: Posterior body region. (Scale bars: A, C-F = 10 μm; B = 30 μm).

Morphological description of Iranian material (Figs. 1-3).

**Figure 4:**
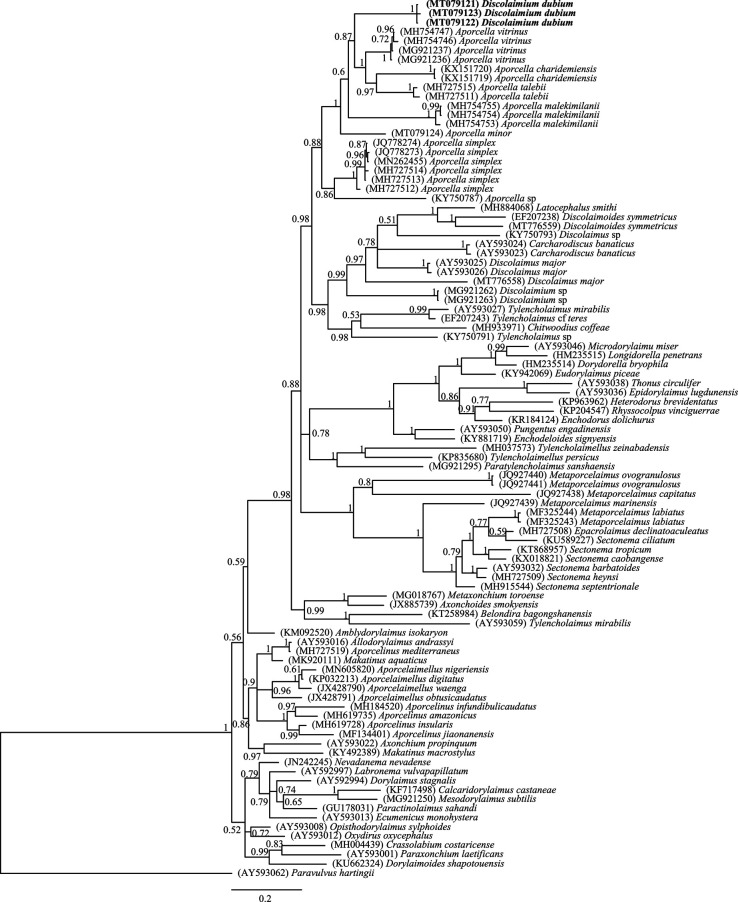
Phylogenetic relationships of *Discolaimium dubium* Das et al., 1969. Bayesian 50% majority rule consensus tree as inferred from D2-D3 expansion segments of 28S rDNA sequence alignments under the GTR + G + I model. Posterior probabilities are given for appropriate clades. Newly obtained sequences are indicated by bold letters.

### Morphometrics

See Table 1.

**Table 1.** Morphometrics of Iranian material of *Discolaimium dubium* Das et al., 1969.

Population	Sufiyan	Teymourlu	Qazi Jahan		Total range	Type
Character	Female	Female	Female	Male	Female	Female
*n*	8	6	6	3	20	25
*L*	1.33 ± 0.04 (1.25-1.39)	1.20 ± 0.06 (1.10-1.31)	1.20 ± 0.03 (1.10-1.30)	1.30 ± 0.08 (1.20-1.40)	1.10-1.40	1.06-1.35
*a*	43.0 ± 2.8 (39-48)	46.2 ± 2.2 (43-49)	44.9 ± 2.7 (41-48)	45.1 ± 2.3 (41.0-48.0)	39-49	40-49
*b*	3.9 ± 0.1 (3.9-4.1)	4.2 ± 0.4 (3.5-4.7)	3.9 ± 1.3 (3.8-4.1)	3.9 ± 0.3 (3.5-4.1)	3.5-4.7	3.5-4.1
*c*	38.0 ± 2.1 (36-43)	34.4 ± 1.3 (34.0-37.0)	38.0 ± 1.2 (37-40)	37.2 ± 3.8 (32.0-42.0)	32-43	33-39
*c′*	1.7 ± 0.1 (1.6-2.0)	1.8 ± 0.2 (1.8-2.0)	1.7 ± 0.04 (1.7-1.8)	1.7 ± 0.2 (1.6-1.9)	1.6-2.0	1.5-2.0
*V*	55.0 ± 1.5 (53-57)	54.0 ± 2.4 (52.0-58.0)	55.0 ± 0.6 (54-56)	–	52-58	53-57
Lip region diam.	9.0 ± 0.5 (8-10)	9.0 ± 0.2 (8.5-9.5)	9.2 ± 0.2 (9.0-9.5)	9.2 ± 0.1 (9.0-9.5)	8-10	10
Odontostyle length	9.4 ± 0.5 (9-10)	8.3 ± 0.7 (7.5-9.5)	9.6 ± 0.1 (9.0-10.0)	9.2 ± 0.04 (9.0-9.5)	7.5-10.5	8-10
Odontophore length	17.0 ± 1.3 (15-19)	15.7 ± 11 (14.5-17.5)	15.4 ± 0.4 (15-16)	15.6 ± 0.3 (15.5-16)	14.5-19	12-15
Guiding ring from ant. end	4.5 ± 0.3 (4-5)	5.1 ± 0.2 (5.0-5.5)	5.1 ± 0.1 (5.0-5.5)	5.0 ± 0.0 (5.0)	4-5.5	?
Neck length	334 ± 10 (318-343)	322 ± 24 (312-350)	318 ± 33 (300-332)	345 ± 15 (331-362)	300-362	320-354
Pharyngeal expansion length	156 ± 2.1 (153-159)	145 ± 12 (127-159)	139 ± 7.7 (131-147)	165 ± 14 (156-181)	127-181	?
Body diam. at neck base	28.0 ± 1.4 (26-30)	26.7 ± 1.2 (25-28)	27.5 ± 0.8 (26-29)	28.3 ± 1.6 (26-30)	25-30	?
mid-body	31.0 ± 1.6 (29-34)	26.7 ± 1.2 (25-29)	27.1 ± 1.4 (25-29)	29.5 ± 3.4 (26-33)	25-34	27
Anus	18.0 ± 0.9 (17-20)	18.7 ± 0.6 (17-19)	19.3 ± 0.9 (19-21)	21.0 ± 0.9 (20-22)	17-22	?
Prerectum length	83 ± 15 (74-103)	85.0 ± 18.3 (65-109)	80.5 ± 7.0 (73-88)	86.0 ± 12 (70-100)	65-109	
Tail length	34.2 ± 1.3 (34-36)	36.2 ± 1.6 (34-38)	33.3 ± 0.6 (32-34)	36.5 ± 1.2 (34-38)	32-38	34
Spicule length	–	–	–	31.0 ± 0.9 (30-32)	–	–
Ventromedian supplements	–	–	–	(2-3)	–	–

**Note:** Measurements in µm, except L in mm.

### Adult

Slender to very slender (*a* = 39-49) nematodes of medium size, 1.10 to 1.40 mm long. Body cylindrical, tapering toward both ends, but more so toward the posterior extremity as the tail is conical. Upon fixation, habitus regularly curved ventrad, to an open C shape in females and nearly J shape in males. Cuticle two-layered, 1 µm thick at anterior region, 1.5 to 2.5 µm in mid-body and 2.5 to 3.5 µm on tail, consisting of thin outer layer bearing fine but conspicuous transverse striation, and thicker inner layer, more appreciable at caudal region. Lateral chord 7 to 10 µm broad, occupying 21 to 28% of mid-body diameter, lacking any differentiation. Lateral pores obscure. Lip region somewhat truncate anteriorly, slightly expanded (but not discoid), that is, slightly wider than its adjoining body, 2.6 to 3.3 times as wide as high and *ca* one-third (26-37%) of body diameter at neck base; lips mostly amalgamated, with weakly protruding papillae. Amphidial fovea cup-like, its aperture occupying 4.5 to 6 µm or more than one-half (50-63%) of lip region diameter. Cheilostom comparatively short and broad, with thick walls. Odontostyle relatively short and robust, 4.1 to 4.6 times as long as wide, nearly equal (0.9-1.0 times) to the lip region diameter, and 0.66 to 0.76% of the total body length; aperture large, 5.5 to 6.5 µm long, occupying more than one-half (59-76%) of the odontostyle length. Guiding ring simple, but conspicuous, situated at 4.5 to 5.5 µm or 0.5 to 0.6 times the lip region diameter from the anterior end. Odontophore rod-like, 1.7 to 2.0 times the odontostyle, lacking any differentiation. Pharynx entirely muscular, consisting of a slender section gradually enlarging into the basal expansion that is 7.7 to 9.5 times as long as wide, 4.5 to 5.5 times the body diameter at neck base and occupies 44 to 46% of the total neck length; gland nuclei located as follows (*n* = 2): DO = 58, 60; DN = 63; S_1_N_1_ = 72, 75; S_1_N_2_ = ?82, S_2_N = 88, 90. Pharyngo-intestinal junction with a rounded conoid cardia 5.5−9.5 × 8.5–12.5 µm, surrounded by intestinal tissue. Tail conical, with rounded tip, ventrally nearly straight, dorsally regularly convex; inner core not reaching the tail tip, therefore a hyaline portion is present occupying 5 to 5.5 µm or 14 to 15% of tail length; caudal pores two pairs, at the middle of tail, one nearly dorsal, other subdorsal.

### Female

Genital system didelphic-amphidelphic, with both branches equally and moderately developed, the anterior 111 to 203 µm long or 9 to 16% of body length, the posterior 108 to 202 µm or 8 to 17% of body length. Ovaries reflexed, relatively small, often not reaching the oviduct-uterus junction, the anterior 52 to 74 µm, the posterior 54 to 90 µm long. Oviduct 44 to 74 µm or 1.3 to 2.1 times the body diameter long, consisting of a slender distal portion made of prismatic cells and a moderately developed *pars dilatata*. A marked sphincter separates oviduct and uterus. Uterus a simple, tube-like structure, 38 to 53 µm or 1.2 to 1.6 times the body diameter long. Vagina extending inwards 11 to 15 µm or *ca* two-fifths (33-43%) of body diameter: *pars proximalis* 7–10 × 8–11 µm, with slightly convergent walls surrounded by weak circular musculature and *pars distalis* 3.5 to 4.5 µm long. Vulva a somewhat posterior, transverse slit. Prerectum 3.0 to 6.6, rectum 1.2 to 1.5 times the anal body diameter long.

### Male

Genital system diorchic, with opposed testes. In addition to the adcloacal pair, located at 8 to 9 µm from the cloacal aperture, there are two or three, widely spaced 13.5 to 16 µm apart, ventromedian supplements, the most posterior of them situated far from the ad-cloacal pair, at 59 to 79 µm. Spicules dorylaimid, regularly curved ventrad and relatively robust, 3.9 to 4.9 times as long as wide; head small, 2.5 to 3 μm long, occupying 8 to 10% of total spicule length, with its dorsal side slightly longer than the ventral one; ventral side bearing visible hump and hollow, median piece occupying 85 to 87% of spicule length. Lateral guiding pieces 8.5 to 9 µm long. Prerectum 2.4 to 4.5, cloaca 1.4 to 1.5 times body diameter at level of cloacal aperture.

### Molecular characterization

Three D2-D3 28S rDNA sequences were obtained, *ca* 800 bp long. Their analysis has allowed the elucidation of evolutionary relationships of the species. The phylogenetic results are presented in Figure 4.

### Distribution

This species has been collected in three locations of East-Azarbaijan province, northwestern Iran: (i) Sufiyan, Roodghat area, Zeinabad village (GPS coordinates: N 38°16′ 49′′E 46° 07′ 18′′, altitude 1598 m a.s.l.), in soil around roots of black cherry trees (*Prunus cerasus* L.); (ii) Osku, Teymourlu (GPS coordinates: N 37°48′ 35′′E 45° 53′ 37′′), collected from rhizosphere of Almond trees (*Prunus dulcis* L.); and (iii) Azarshahr, Qazi Jahan village (GPS coordinates: N 37°46′ 27′′E 45° 55′ 51′′), from the rhizosphere of walnut trees (*Juglans regia* L.).

## Discussion

### Comparison of Iranian specimens with type material of *D. dubium* and its closely related species from the genus *Aporcella*

The description of *D. dubium* by Das et al. (1969) was not excessively detailed, but available information is enough for comparative purposes. Table 1 shows that the most relevant morphometrics of Iranian nematodes and type material (consisting of specimens from at least three European enclaves, see above) are nearly identical, with totally coincident or widely overlapping ranges. General description, in particular the morphology of pharynx, genital system, and caudal region, are identical, as well. Thus, there is no doubt that the Iranian material belongs to the species. The above mentioned description therefor confirms the original one and provides an updated morphometric and morphological characterization of the species. Morphometrical characters of Iranian population of *D. dubium* and closely related species in the genus *Aporcella* are shown in Table 2.

**Table 2.** Main morphometrics of Iranian material of *Discolaimium dubium* Das et al., 1969 and its seven close species of the genus *Aporcella*.

									Characters								
Species	*n*	*L*	*a*	*b*	*c*	*c′*	*V*	Lrd^a^	Odont.	Neck	Ph.exp.	Prerect.	Tail	Spicul.	Ve.sup.	Geog.dis.	Ref^b^
*D. dubium*	20♀♀	1.10-1.39	39-49	3.5-4.7	34-43	1.6-2.0	52-58	8.0-10	7.5-10	300-350	127-159	65-109	32-38	–	–	Iran	1
	3♂♂	1.20-1.40	41-48	3.5-4.1	32-42	1.6-1.9	–	9.0-9.5	9.0-9.5	331-362	156-181	700-100	34-38	30-32	2-3		
*A. adriaani*	7♀♀	1.23-1.49	23-30	3.5-4.1	42-58	0.8-1.1	53-57	12-15	14-16	320-380	183	63-179	21-30			South Africa	2
*A. debruinae*	2♀♀	2.14-2.20	40	4.2-4.5	65-76	0.9-1.0	49-50	15-16	16	480-510	–	191	29-33	–	–	Botswana	2
	2♂♂	2.12-2.23	42-43	4.4-4.8	76-93	0.8-0.9	–	16-17	14-15	460-480	–	–	24-28	57-61	5		
*A. malekimilani*	23♀♀	1.90-2.70	32-55	4.0-5.0	41-58	1.2-1.7	55-61	14-17	14-15	476-619	300-375	105-178	44-56	–	–	Iran	3
*A. minor*	8♀♀	1.05-1.15	27-32	3.3-3.6	33-36	1.4-1.6	58-61	11-13	12.5-14	293-325	128-144	38-55	29-33	–	–	Iran	3
*A. talebii*	13♀♀	1.66-2.02	27-37	3.7-4.4	37-47	1.1-1.4	52-59	15-17	14-18	412-484	209-231	55-84	40-50	–	–	Iran	3
*A. tropica*	9♀♀	1.44-1.76	39-50	3.8-4.9	63-82	0.8-1.0	55-62	–	12-14	333-368	–	–	22-24	–	–	India	2
	9♂♂	1.60-1.80	44-54	4.2-4.9	60-77	1.0	–	–	–	–	–	–	22-27	42-47	3-5		
*A. vitrinus*	32♀♀	1.20-1.86	30-47	4.0-5.1	45-77	0.9-1.4	48-53	11-13	12-13	333-374	149-185	44-132	25-37	–	–	Spain	3
	♂♂	1.47	?	?	48	1.0	–	13	12	?	?	107	31	43	6		
	15♀♀	1.4-2.0	26-41	3.4-4.5	50-65	0.9-1.1	50-56	12-13	12-15	363-406	175-199	72-109	22-31	–	–	Iran	

**Notes:** All measurements in µm, except L in mm. ^a^Abbreviations: Lrd, lip region diameter; Odont., odontostyle length; Ph.exp., pharyngeal expansion length; Prerect., prerectum length; Spicul., spicule length; Ve.sup., number of ventromedian supplements; Geog.dis., geographical distribution. ^b^References: 1 – Present paper. 2 – Álvarez-Ortega et al. (2013). 3 – Vazifeh et al. (2020).

### Proposal of a new identity for *D. dubium*

Since its original description, *D. dubium* has been regarded as an atypical species within the genus *Discolaimium* (Das et al., 1969; Patil and Khan, 1982). This study of Iranian specimens confirms this opinion as several traits (pharynx entirely muscular and enlarging gradually, anterior position of DO, absence of lateral gland bodies) are not usual in members of this genus. Besides, the odontostyle aperture is appreciably longer than one-half of the total length, a typical feature of aporcelaims, the members of the family Aporcelaimidae Heyns, 1965.

Molecular analyses of LSU rDNA sequences of three Iranian specimens of *D. dubium* sheds some light on the identity of this species. Their evolutionary relationships, as observed in the tree provided in Figure 4, reveal that these sequences form part of a highly supported (98%) large clade including several taxa, all of them having in common the absence of *pars refringens vaginae*, so confirming previous findings (Álvarez-Ortega and Peña-Santiago, 2016; Álvarez-Ortega et al., 2013; Imran et al., 2019; Naghavi et al., 2019). The most relevant result is, however, that *D. dubium* sequences form a very robust (100%) subclade with members of the genus *Aporcella* (Andrássy, 2002). Other discolaims constitute another robust (98%) subclade. This strongly supports the membership of *D. dubium* in *Aporcella*, which is totally compatible with the updated morphological characterization of the species above provided. Actually, in its general morphology, *D. dubium* resembles several conical-tailed members of *Aporcella* from which it can be easily distinguished in several relevant morphometrics (see below). Consequently, it is transferred to *Aporcella* as *A. dubia* (Das et al., 1969) comb. n.

### Separation of *A. dubia* from similar *Aporcella* species

For comparative purposes and taken into consideration the results provided in the present study, *A. dubia* is characterized by its 1.10 to 1.40 mm long body, cuticle bearing fine but perceptible transverse striation, lip region offset by constriction and 8 to 10 µm wide, odontostyle 7.5 to 10.5 µm long with aperture occupying 59 to 76% of total length, neck 300 to 362 µm, pharyngeal expansion 127 to 181 µm long or 44 to 46% of the total neck length, female genital system didelphic-amphidelphic, uterus simple and 38 to 53 µm or 1.2 to 1.6 times the body diameter long, vulva a transverse slit (*V* = 52-58), tail conical (32-38 µm, *c* = 32-43, *c′* = 1.6-2.0) with rounded tip and a hyaline portion occupying 14 to 15% of tail length, spicules 30 to 32 µm long, and two or three widely spaced, ventromedian supplements with hiatus.

By having slender body (*a*-ratio ≥ 39), short odontostyle (< 17 µm long) and comparatively short pharyngeal expansion (up to 54% of the total neck length), *A. dubia* resembles *A. debruinae* Álvarez-Ortega et al., 2013, *A. minor* Vazifeh et al., 2020, and *A. tropica* (Álvarez-Ortega et al., 2013; Jana and Baqri, 1981). It differs from both *A. debruinae* and *A. tropica* in its smaller general size (body length 1.06-1.40 mm, *n* = 45 *vs* 1.44-1.88 and 2.12-2.23 mm, respectively), narrower lip region (8-10 *vs* 15-16 and 11.5-13 µm, respectively), shorter odontostyle (7.5-10.5 *vs* 11-14 and 14-16 µm, respectively) and much shorter spicules (30-32 *vs* 42-47 and 57-61 µm, respectively). From *A. minor*, a similar species recently described from Iran too, in its more slender body (*a* = 39-49 *vs* *a* = 27-32, n = 9), narrower lip region (8-10 *vs* 11-13 µm), shorter odontostyle (7.5-10.5 *vs* 12.5-14 µm), and more anterior vulva (*V* = 52-58 *vs* *V* = 58-61).

### Remarks

The transference of *D. dubium* to *Aporcella* brings up the taxonomy of the large clade morphologically characterized by the absence of *pars refringens vaginae* and robustly supported by molecular data. This clade consists of representatives of at least three traditional dorylaimid families, namely Aporcelaimidae, Qudsianematidae Jairajpuri, 1965, and Tylencholaimidae Filipjev, 1934. Leaving aside the tylencholaims (Tylencholaimidae), in general easily distinguishable thanks to the leptonchid (= tylencholaimid) nature of their cuticle, the morphological separation of aporcelaims (Aporcelaimidae, represented by *Aporcella* sequences) and discolaims (Qudsianematidae, Discolaiminae Siddiqi, 1969, represented by *Carcharolaimus, Discolaimus* and *Discolaimoides* sequences) of this clade becomes controversial and problematic. The results obtained in the present contribution suggest that some species currently classified under *Discolaimium* (and perhaps other species under *Discolaimoides* too) might belong to *Aporcella* and that, as consequence, a re-evaluation and/or re-definition of these genera are recommendable and necessary.
